# Sphingosine Kinase 1 and Sphingosine-1-Phosphate Signaling in Colorectal Cancer

**DOI:** 10.3390/ijms18102109

**Published:** 2017-10-08

**Authors:** Yonghua Bao, Yongchen Guo, Chenglan Zhang, Fenghua Fan, Wancai Yang

**Affiliations:** 1 Institute of Precision Medicine, Jining Medical University, Jining 272067, China; baoyonghua2005@126.com (Y.B.); guoyongchen2005@126.com (Y.G.); 2 Department of Nursing, Health Professional College of Heilongjiang Province, Beian 164000, China; Chenglanzhang2016@126.com (C.Z.); fenghuafan2016@126.com (F.F.); 3 Department of Pathology, University of Illinois at Chicago, Chicago 60612, IL, USA

**Keywords:** sphingosine kinase 1, sphingosine-1-phosphate, colorectal cancer

## Abstract

Sphingosine kinase 1 (Sphk1) is a highly conserved lipid kinase that phosphorylates sphingosine to form sphingosine-1-phosphate (S1P). Growing studies have demonstrated that Sphk1 is overexpressed in various types of solid cancers and can be induced by growth factors, cytokines, and carcinogens, leading to the increase of S1P production. Subsequently, the increased Sphk1/S1P facilitates cancer cell proliferation, mobility, angiogenesis, invasion, and metastasis. Therefore, Sphk1/S1P signaling plays oncogenic roles. This review summarizes the features of Sphk1/S1P signaling and their functions in colorectal cancer cell growth, tumorigenesis, and metastasis, as well as the possible underlying mechanisms.

## 1. Introduction

Sphingosine kinase 1 (Sphk1) is a highly conserved lipid kinase that functions to phosphorylate sphingosine to form sphingosine-1-phosphate (S1P). The Sphk1/S1P-signaling pathway has exerted important physiological and pathophysiological functions. Growing evidence has demonstrated that Sphk1/S1P signaling plays oncogenic roles and promotes carcinogenesis and progression. For instance, the increased Sphk1/S1P facilitates cancer cell proliferation, mobility, angiogenesis, invasion, and metastasis. Therefore, Sphk1/S1P signaling could be a useful target for preventions and therapy. Although increasing studies have reported the important roles of Sphk1/S1P signaling in colorectal cancers, there is a lack of systemic summarization. This review summarizes the features of Sphk1/S1P signaling and their functions in colorectal cancer cell growth, tumorigenesis, and metastasis, inhibitors of Sphk1/S1P signaling, as well as the possible underlying mechanisms.

## 2. Characterization and Physiological Functions of Sphingosine Kinase and Sphingosine-1-Phosphate-Signaling Pathway

Sphingosine kinases are highly conserved lipid kinases that contain five conserved domains and phosphorylate sphingosine to form sphingosine-1-phosphate (S1P). Sphingosine kinase 1 (Sphk1) was originally purified from rat kidney, and murine Sphk1 was then cloned and functionally characterized based on the peptide sequences of rat Sphk1 [1,2]. The human sphingosine kinase encodes a 384-amino acid protein with 85% identity to murine Sphk1 at the amino acid level [3]. It has three calcium/calmodulin-binding consensus sequences, several potential protein kinase phosphorylation sites, and no trans-membrane domains. Sphk1 was widely expressed with higher levels in liver, kidney, lung, heart, spleen, brain, and skeletal muscle. Sphk1 is mainly present in the cytosol and needs to be translocated to the plasma membrane to be activated [4].This translocation then enhances S1P production in the local environment, which in turn activates five G protein-coupled receptors (S1PR1-5)-a process known as “inside-out signaling” by S1P [5]. Sphk1 can be stimulated by numerous external stimuli, such as growth factors and their receptors (e.g., Platelet derived growth factor(PDGF), Vascular endothelial growth factor (VEGF), Nerve growth factor (NGF), and Epidermal growth factor (EGF)), ligands for G-protein coupled receptors, transforming growth factor β (TGF-β), and tumor necrosis factor α (TNF-α), a pro-inflammatory cytokine. TNF-α and other cytokines stimulate Sphk1, leading to the activation of the transcription factor nuclear factor-κB (NF-κB) [6]. The activation of Sphk1 can also activate oncogenic-signaling elements; e.g., protein kinase C/Ras, extracellular signal regulated kinase (ERK), mitogen-activated protein kinase (MAPK), resulting in cell growth and angiogenesis, with vascular endothelial growth factor (VEGF)-induced DNA synthesis [7,8]. The activity and subcellular localization of Sphk1 are also influenced by its interaction with adaptor proteins such as RPK118—a protein related to protein kinase A anchoring protein, and mast cell protein tyrosine kinases Lyn and Syk [4].

The second type of mouse and human sphingosine kinase (mSphk2 and hSphk2) has also been well characterized. mSphk2 and hSphk2 encode proteins of 617 and 618 amino acids, respectively, which are larger than Sphk1 protein. Sphk2 mRNA expression has a strikingly different tissue distribution from that of Sphk1 and appears later in embryonic development. In vitro studies showed that expression of Sphk2 results in elevated S1P production [9]. However, the biological functions of Sphk2 are largely different from Sphk1: Sphk1 acts as an oncogene and Sphk2 exerts tumor suppressive roles in colorectal carcinogenesis and progression. For example, Sphk1- but not Sphk2- is responsible for S1P export from breast cancer cells and S1P is linked to inflammation and cancer incolitis-associated cancer progression, while Sphk2 is linked to sensitization of human colon cancer cells to sodium butyrate-induced apoptosis; loss of Sphk1- not Sphk2- attenuates the development of intestinal inflammation and colitis-associated colorectal carcinogenesis [10,11,12]. This review focuses on Sphk1/S1P signaling.

TNF-α receptor-associated factor 2 (TRAF2) is one of the major mediators of TNF receptor super family signaling to activate NF-κB, c-Jun N-terminal kinase (JNK), and anti-apoptosis, and the TRAF2-binding motif of Sphk1 facilitates the interaction between TRAF2 and Sphk1, and results in the Sphk1 activation, which is important for the suppression of TNF signaling-mediated apoptosis and shows a critical role for Sphk1/TRAF2-mediated signal transduction to the activation of NF-κB and anti-apoptosis [6,13]. These findings suggest that Sphk1 plays an important role in cell survival and its activation enhances cell survival in response to TNF-α, and that Sphk1 may be a component of the signal transduction pathway downstream of the TNF receptor and TRAF2.

One of the major functions of Sphk1 is to phosphorylate sphingosine and form the pleiotropic lipid mediator sphingosine-1-phosphate (S1P). S1P is mainly distributed in the high-density lipoprotein (HDL) fraction in plasma and serum, and can interact with endothelial cells. Therefore, S1P plays a role in endothelial cell migration, proliferation, and angiogenesis, promoting blood vessel formation through interaction with signaling by angiogenic growth factor VEGF [8].

S1P also plays important roles in immunity and inflammation [14,15]. S1P has been shown to be a major regulator for the mononuclear phagocytes and lymphocytes for differentiation, migration, circulation, tissue homing patterns, and chemotactic responses to chemokines [16]. Moreover, because T and B lymphocytes as well as endothelial cells express distinctive profiles of G protein-coupled receptors for S1P, activation of T and B lymphocytes by various immunological stimuli also suppresses expression of S1P [17].

## 3. Sphk1/S1P Signaling in Cell Growth, Cell Cycle, and Tumorigenesis

S1P/Sphk1 has been implicated as an oncogenic signaling pathway to regulate diverse cellular functions, including cell growth, proliferation, migration, and survival [18]. Growing evidence has shown that increased expression of Sphk1 and production of S1P promote cell growth and enhance the cell cycle transition from G1 to S phase, causing malignant transformation and anti-apoptosis [19]. For instance, increased expression of Sphk1 markedly increased S1P levels in murine NIH 3T3 fibroblasts and human HEK293 cells. Moreover, increased Sphk1 activity was sufficient to promote NIH 3T3 fibroblast growth in soft agar and tumor formation in nude mice, expedite the G1/S transition, and increase DNA synthesis as well as the proportion of cells in the S phase of the cell cycle, which was through activation of Ras and extracellular signal-regulated protein kinases 1 and 2 (ERK1/2) signaling, suggesting a potential oncogenic function [19]. Transient or stable overexpression of Sphk1 in NIH 3T3 fibroblasts or HEK293 cells protected against apoptosis induced by serum starvation [19]. In addition, enforced overexpression of Sphk1 in human breast cancer cell line MCF-7 cells enhanced cell proliferation and resistance to tamoxifen-induced cell growth arrest and apoptosis. In contrast, inhibition of Sphk1 activity by either specific pharmaceutical inhibitors or dominant-negative Sphk1 restored the antiproliferative and proapoptotic effects of tamoxifen, and inhibited MCF-7 transformation and malignant ability both in vitro and in nude mice [20]. Furthermore, Sphk1 is frequently overexpressed in a variety of solid tumors, suggesting an important oncogenic role [21]. In addition, Sphk1/S1P signaling is associated with cancer metastasis, chemoresistance, and survival of patients with colorectal cancers [21,22] and neuroblastoma [23]. Recent studies have also shown that Sphk1/S1P signaling is a preventive and therapeutic target for cancers [24].

MicroRNAs (miRNAs) have been reported as oncogenes or tumor suppressors, and thus exhibited significant roles in carcinogenesis and progression through regulation of oncogenic or suppressive-signaling pathways, stem cells, epithelial–mesenchymal transition (EMT), and metastasis [25,26]. Several lines of evidence have also demonstrated that Sphk1 is a downstream target of microRNAs and acts as oncogene in promoting tumorigenesis and progression. For example, miRNA-124 has been shown downregulated in various types of solid cancers, and acts as a tumor suppressor by inhibiting cancer cell proliferation, invasion, and metastasis in the head and neck squamous cell carcinoma, osteosarcoma, ovarian, and gastric cancers by targeting Sphk1 signaling [27,28,29]. MiRNA-101 inhibited colorectal cancer cell proliferation by targeting Sphk1 [30]. In addition, miRNA-613 targets Sphk1 and inhibits bladder cancer cell proliferation [31], and miR-506 suppresses liver cancer angiogenesis by targeting Sphk1 mRNA [32], downregulated miR-506 expression facilitates pancreatic cancer progression and chemoresistance via Sphk1/Akt/NF-κB signaling [33]. On the other hand, long non-coding RNA HULC (Hepatocellular carcinoma up-regulated long non-coding RNA) could upregulate Sphk1 and promote liver cancer angiogenesis [34]. Moreover, Sphk1 is a direct target of miR-659-3p in colorectal cancer cells, and it is negatively regulated by miR-659-3p [35]. The miR-659-3p/Sphk1 is involved in the regulation of chemotherapy responses of colorectal cancer cells in vivo [35], in which expression of miR-659-3p was significantly reduced in cisplatin-resistant colorectal cancer (CRC) samples, compared to cisplatin-sensitive CRC samples; in contrast, compared to cisplatin-sensitive colorectal cancers, the expression of Sphk1 was significantly increased in cisplatin-resistant samples [35]. As reported, circulating miRNA in body fluid could be detected and used for the diagnosis of lung cancers [36], liver cancers [37] and other cancers [38]. Sphk1/S1P is also circulating in body fluid and is possible to be detected. However, the diagnostic value of circulating Sphk1/S1P, unlike miRNAs, needs further investigation since there has no acceptable reference levels, although it has been reported that circulating S1P and erythrocyte Sphk1 activity could be a biomarkers for early prostate cancer detection [39].

## 4. Sphk1/S1P in Chronic Colitis and Colorectal Carcinogenesis

Previous studies have demonstrated that Sphk1 expression levels are increased in the colons of patients with ulcerative colitis (UC) or colorectal cancers [22,40], and the increased expression of Sphk1 and S1P is associated with poor outcomes. The recent study has also reported that Sphk1 is overexpressed in CRC cell lines besides the upregulation in CRC tissues, and the upregulation of Sphk1 is significantly correlated with lymph node metastasis, liver metastasis, and advanced TNM stage [40]. In vitro, knockdown of Sphk1 in colorectal cancer cells results in inhibition of cancer cell proliferation, migration, and invasion, which is through increasing E-cadherin expression and decreasing vimentin expression [40]. Whereas, increased expression of Sphk1 in colon cancer cell line HT-29 cells enhances tumor growth in nude mice [41]. Furthermore, overexpression of Sphk1 in intestinal epithelial cells via a transgenic strategy significantly enhances azoxymethane (AOM)-induced colon tumor formation in mice [41].

**Figure 1:**
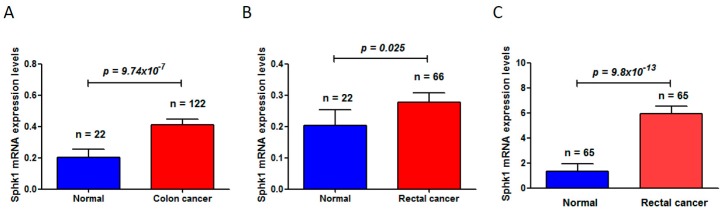
Sphingosine kinase 1 (Sphk1) mRNA levels were significantly increased in colorectal cancers, compared to the matched adjacent non-cancer adjacent tissues. (**A**) Sphk1 was increased in colon cancers (The Cancer Genome Atlas, TCGA, colorectal cancer dataset); (**B**) Sphk1 was increased in rectal cancers (TCGA colorectal cancer dataset); (**C**) Sphk1 was increased in rectal cancers (Oncomine dataset). “*n*” is the number of subjects studied in each group.

Through deep mining of the published dataset in The Cancer Genome Atlas (TCGA) (available online: https://cancergenome.nih.gov/) [42,43], we found that Sphk1 is significantly increased in colon cancers (normal mucosa 0.20 ± 0.05 vs. 0.41 ± 0.42 in colon cancer (mean ± standard deviation), *p* = 9.74 × 10^−7^) (Figure 1A) and in rectal cancers (normal mucosa 0.20 ± 0.05 vs. 0.28 ± 0.24 in rectal cancers (mean ± standard deviation), *p* = 0.025) (Figure 1B). Another dataset from the Oncomine (available online: www.oncomine.org) [44] also showed a significant increase of Sphk1 in rectal cancers (normal mucosa 1.355 ± 0.56 vs. rectal cancer 5.94 ± 4.59 (mean ± standard deviation), *p* = 9.8 × 10^−13^) (Figure 1C). More studies in rodent models have also demonstrated that *Sphk1* is overexpressed in the tumors of the *Apc^Min/+^* mouse model of colon cancers and plays critical roles in intestinal tumorigenesis and progression [45]. Increased expression levels of Sphk1 and S1P in colorectal cancers and in the plasma were also observed in mouse models with the treatment of carcinogen azoxymethane (AOM) followed by chronic colitis induced by oral administration of dextran sodium sulfate (DSS) [46]. However, genetic deletion of *Sphk1* reduced AOM/DSS-induced development of chronic colitis and intestinal tumor formation in the mice [46], leading to the downregulation of NF-κB and production of NF-κB-regulated proinflammatory cytokines tumor necrosis factorα (TNF-α) and Interleukin 6 (IL-6), and signal transducer and activator of transcription 3 (Stat3), which are key mediators in colitis and in the development of colitis-associated colorectal cancer (CAC) [47,48].

Recent work has revealed that the link between inflammation and cancer is through the Sphk1/S1P/S1P receptor 1 (S1PR1) axis that contributes to the NF-κB/IL-6/Stat3 amplification loop. Similar to Sphk1, S1PR1 is also upregulated in AOM/DSS-induced murine models of colitis and CAC [22]. These findings strongly suggest that upregulation of Sphk1 in intestinal epithelial cells and increased formation of S1P during intestinal inflammation and colorectal malignant transformation results in the activation of S1PR1 and the downstream activation of Src or JAK, which can phosphorylate and activate Stat3 [49]. Stat3 then regulates the expression of many genes involved in both cell survival and proliferation, including *S1PR1* [12,22,50]. Moreover, Sphk1 and S1P are also involved in activation of NF-κB by TNF-α. This leads to the recruitment of myeloid cells that produce IL-6 and TNF-α—pro-inflammatory cytokines important for the progression of CAC [47,48,51]. Therefore, in intestinal epithelial cells, NF-κB regulates Stat3 activation, the cytokines (e.g., TNF-α and IL-6) stimulate Sphk1 and increase its expression [15], leading to the activation and upregulation of both Stat3 and NF-κB via the S1P/S1PR1-signaling pathway. Therefore, increased expression of Sphk1 and S1P and the activation of S1PR1, play an essential role in maintaining persistent activation of NF-κB and Stat3, leading to the development of chronic intestinal inflammation and its malignant transformation to CAC.

Most recently, we have found that there is a regulatory interaction between PRSS8 (protease serine 8) and Sphk1/S1P/Stat3/Akt signaling [52]. PRSS8 is a membrane-anchored serine protease prostasin, and is overexpressed in epithelial cells of various tissues; it is also involved in terminal epithelial differentiation [53,54,55]. We found that PRSS8 expression was significantly reduced in colorectal cancers, and the decreased expression of PRSS8 was associated with clinical stages, poor differentiation, and poor outcomes. However, enforced expression of PRSS8 led to the inhibition of colorectal cancer cell proliferation and retarded cancer cell growth in nude mice. Mechanistically, PRSS8 negatively correlated with Sphk1 in both a Sphk1 knockout mouse model and human colorectal cancers. Moreover, co-immunoprecipitation assay showed that PRSS8 physically bonds to Sphk1 proteins, and co-localized expression showed cytoplastic and nuclear co-expression of these two proteins [52]. These findings strongly suggest the crosstalk between PRSS8 and Sphk1/S1PStat3 signaling, and opposite functions (i.e., tumor inhibition by PRSS8 and tumor promotion by Sphk1/S1P/Stat3 signaling).

## 5. Sphk1/S1P and Cancer Metastasis

It is known that Sphk1 is overexpressed in colorectal cancer tissues and cell lines, and the upregulation of Sphk1 is well correlated poor survival, mainly due to metastasis to lymph node, liver, and other organs. Indeed, one study showed that colon cancer with metastasis exhibited higher expression of Sphk1/S1P than those without metastasis, and another study showed that the expression levels of Sphk1, the focal adhesion kinase (FAK) pathway, intercellular adhesion molecule 1 (ICAM1), and vascular cell adhesion molecule 1 (VCAM1) were higher in colorectal cancers in comparison with their adjacent non-cancer tissues [56,57]. Moreover, the expression of Sphk1 was significantly correlated with the expression of FAK or p-FAK, and the co-expression of Sphk1, FAK, and p-FAK was significantly associated with colorectal cancer histopathological grades, Dukes’ stages, lymph node metastasis, and distant metastasis [40,56]. In vitro, increased expression of Sphk1 promoted colon cancer cell viability and invasiveness, but suppressed cell apoptosis. In contrast, knocking down SphK1 by shRNA targeting human Sphk1 suppressed cancer cell viability and invasiveness, but facilitated cell apoptosis. The expression levels of FAK, p-FAK, ICAM1, and VCAM1 were also upregulated with the overexpression of Sphk1 but downregulated with the reduction of Sphk1 in colon cancer cells [57]. These findings indicate that Sphk1 plays a critical role in colorectal cancer initiation and progression in vivo and regulates colon cancer cell proliferation, apoptosis, and invasion in vitro, which was through FAK pathway, ICAM-1, and VCAM.

It is known that EMT is a pre-metastasis event in cancer progression. The in vitro studies have demonstrated that inhibition of Sphk1 by SKI-II (Sphk1 inhibitor) or PF-562271 (focal adhesion kinase, FAK, inhibitor) led to the reduction of cell migration, and to the downregulation of EMT-related proteins Slug, vimentin, and N-cadherin, but to the upregulation of E-cadherin [56,57,58]. These findings suggest that Sphk1 is involved in modulating the EMT process and the expression of EMT-related molecules and cell migration through the FAK/p-FAK pathway.

Cancer metastasis and neovascularization could be caused by hypoxia (reduction in the normal level of tissue oxygen tension), in which the activation of the transcription factor hypoxia-inducible factor 1α (HIF-1α) plays an important role. Recent study has revealed the Sphk1/S1P pathway as a new modulator of HIF-1α activity under hypoxic conditions [58]. Under low oxygen tension, Sphk1 activity is quickly stimulated by the production of reactive oxygen species, and intracellularly S1P or S1PR triggers HIF-1α regulator Akt/Glycogen synthase kinase 3β (GSK3β) signaling activation; the latter has been known as one of the key signalings in colorectal carcinogenesis and metastasis. In contrast, reduced expression or enzymatic activity of Sphk1 causes the downregulation of Akt/GSK3β signaling, resulting in the degradation of HIF-1α by tumor suppressor protein von Hippel-Lindau (pVHL)-mediated ubiquitination.

## 6. Sphk1/S1P as a Target for Chemoprevention and Therapy

Numerous evidence has suggested Sphk1 as a promising target for cancer therapy for the following reasons: Sphk1 is overexpressed in various types of tumors and acts as an oncogene, and Sphk1 can be induced by growth factors, cytokines, mitogens, and carcinogens, and subsequently leads to the increases of S1P; in turn, S1P potentially stimulates cancer angiogenesis, mobility, invasion, and metastasis. Sphk1 upregulation is also linked to resistance to chemotherapy and radio-therapy. Therefore, inhibition of Sphk1 will reduce S1P production and increase sphingosine, promoting cancer cells’ death, which has been supported by several in vitro studies; for example, downregulation of Sphk1 by small interfering RNA targeting human Sphk1 has been found to promote apoptosis in many types of cancer cells, including colon, breast, prostate, glioblastoma, and leukemia, and the increased apoptosis triggered by Sphk1 siRNA was associated with the activation of mitochondrial pathway, including the increase of caspases activation and the release of cytochrome C and sphingosine [24]. The specific inhibitor SKI-II (4-(4-(4-chloro-phenyl)-thiazol-2-ylamino)-phenol) could inhibit cancer cell growth in nude mice by oral administration [59]. In addition, the Sphk1 inhibitor N′-(3-(benzyloxy) benzylidene)-3,4,5-trihydroxybenzohydrazide also decreased expression of interleukin IL-6 and cyclooxygenase-2 (COX-2) and significantly inhibited ulcerative colitis—a precancerous lesion of colorectal cancer—in a DSS-induced rodent model [60]. 

A recent study has also reported that PF-543—a novel Sphk1 inhibitor—exerted potent anti-proliferative and cytotoxic effects against a panel of established colorectal cancer cells lines (e.g., HCT-116, HT-29, and DLD-1 cells) and primary human colorectal cancer cells through a mitochondrially programmed necrosis pathway, but not apoptosis [61]. Its sensitivity was negatively associated with Sphk1 expression level in the CRC cells. In vivo, intravenous injection of PF-543 significantly suppressed HCT-116 xenograft growth in severe combined immuno-deficient (SCID) mice and significantly improved mice survival [61].

The most commonly used pharmacological inhibitors are sphingosine analogues (especially *N*,*N*-dimethylsphingosine, DMS, and DL-threo-dihydrosphingosine, DHS); both are potent competitive inhibitors of Sphk1 [24], leading to cancer cell growth inhibition in vitro and in nude mice, and enhanced apoptosis in the cancer cells of colorectal, breast, prostate, gastric, and lung. Moreover, the reduction of Sphk1 restored cancer cells’ sensitization to chemotherapy and radiotherapy in vitro. A most recent study showed that targeting of Sphk1 was able to induce caspase-dependent cell death in acute myeloid leukemia (AML) cell lines, primary AML patient blasts, and isolated AML patient leukemic progenitor/stem cells, and administration of Sphk1 inhibitors to orthotopic AML patient-derived xenografts could suppress tumor burden and prolong overall survival without affecting murine hematopoiesis [62]. The analogue of sphingosine, FTY720 (Fingolimod), is an Food and Drug Administration (FDA)-approved immunomodulator and has been shown effective in vitro and in vivo cancer models through functional antagonism of S1PR1 and inhibition Sphk1 [24], suggesting a potential therapeutic role in cancer patients.

## 7. Conclusions and Perspectives

**Figure 2:**
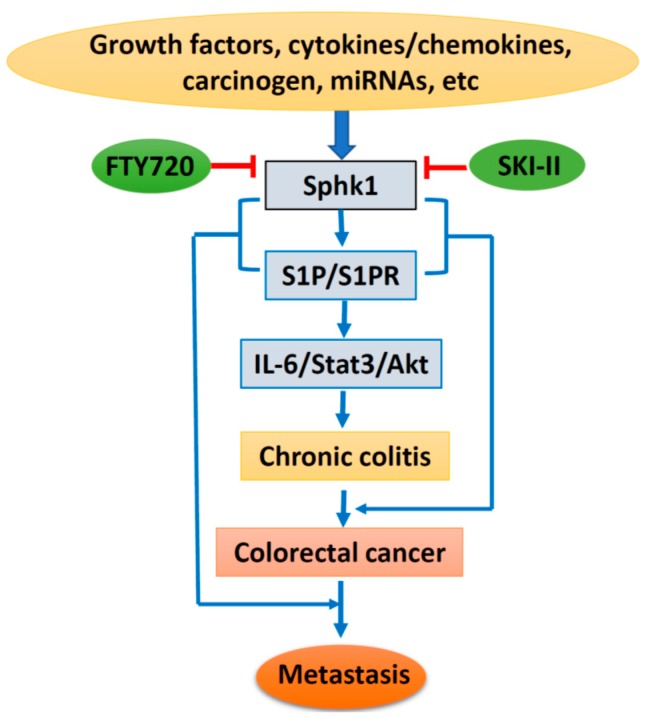
Schematic diagram of Sphk1/sphingosine-1-phosphate (S1P) signaling in colorectal carcinogenesis and progression. IL-6, interleukin 6; State3, signal transducer and activator of transcription 3; Akt, protein kinase B; SKI-II, sphk1 inhibitor.

Based on the above, lipid kinase Sphk1 and the production of S1P and the Sphk1/S1P/S1PR1-signaling pathway could be stimulated by growth factors, cytokines, and carcinogens, with additional regulation of miRNAs. Sphk1/S1P signaling causes chronic colitis and malignant transformation through activation of the IL-6/Stat3/Akt pathway, and further enhances cancer metastasis through interaction with EMT-related proteins. However, specific inhibitors targeting Sphk1 could suppress Sphk1/S1P signaling and inhibit colorectal carcinogenesis and progression (Figure 2). Therefore, targeting the Sphk1/S1P pathway could be a novel strategy for cancer prevention and therapy. However, more drug-like specific Sphk1 inhibitors are highly in demand, and clinical trials are needed for final clinical implication.

## Abbreviations

Sphk1	Sphingosinekinase 1
S1P	Sphingosine-1-phosphate
S1PR1	S1P receptor 1
CRC	Colorectal cancer
CAC	Colitis-associated colorectal cancer
UC	Ulcerative colitis
EMT	Epithelial-mesenchymal transition
ERK	Extracellular signal regulated kinase
MAPK	Mitogen-activated protein kinase
NF-κB	Nuclear factor-κB
HDL	High-density lipoprotein
TNF-α	Tumor necrosis factor α
TRAF2	TNF receptor-associated factor 2
JNK	c-Jun N-terminal kinase
FAK	Focal adhesion kinase
VEGF	Vascular endothelial growth factor
TCGA	The Cancer Genome Atlas
MiRNAs	MicroRNAs
AOM	Azoxymethane
DSS	Dextran sodium sulfate
PRSS8	Protease serine 8
ICAM1	Intercellular adhesion molecule 1
VCAM1	Vascular cell adhesion molecule 1
PDGF	Platelet derived growth factor
PDGF	Platelet derived growth factor
NGF	Nerve growth factor
VEGF	Vascular endothelial growth factor
EGF	Epidermal growth factor
TGF-β	Transforming growth factor β
HIF-1α	Hypoxia-inducible factor 1α
GSK3β	Glycogen synthase kinase 3β
COX-2	Cyclooxygenase-2
IL-6	Interleukin 6
Stat3	Signal transducer and activator of transcription 3
Akt/PKB	Protein kinase B
SCID	Severe combined immuno-deficient
DMS	*N*,*N*-dimethylsphingosine
DHS	DL-threo-dihydrosphingosine
AML	Acute myeloid leukemia
FDA	Food and drug administration
SKI-II	Sphk1 inhibitor
